# Loss of IL‐4Rα–mediated PI3K signaling accelerates the progression of IgE/mast cell–mediated reactions

**DOI:** 10.1002/iid3.80

**Published:** 2015-09-17

**Authors:** Jane Sledd, David Wu, Richard Ahrens, Jeebong Lee, Lisa Waggoner, Ying Ting Tsai, Yui‐Hsi Wang, Simon P. Hogan

**Affiliations:** ^1^ Divisions of Allergy and Immunology and of ImmunobiologyDepartment of PediatricsUniversity of Cincinnati College of MedicineCincinnati Children's Hospital Medical Center3333 Burnet AvenueCincinnatiOH 45229

**Keywords:** food‐induced anaphylaxis, IgE and mast cells, interleukin 4 (IL‐4) receptor (IL‐4R) chain

## Abstract

Clinical and experimental evidence indicate that polymorphisms within the interleukin 4 (IL‐4) receptor (IL‐4R) chain are sufficient for altered strength of IL‐4/IL‐13 signaling, leading to an exaggerated allergic inflammatory response and increase susceptibility to allergic phenotypes. In the present study, we show that ablation of IL‐4Rα–induced phosphatidylinositol 3‐kinase (PI3K) activating signal by germline point mutation within the IL‐4Rα motif (Y500F) did not alter susceptibility to IgE‐mediated, food‐induced experimental anaphylaxis. Moreover, diarrhea occurrence, antigen‐specific IgE and intestinal mastocytosis were comparable between WT and IL‐4Rα^Y500F^ mice. However, mice unable to stimulate IL‐4Rα–mediated PI3K signaling had accelerated disease progression. Notably, the accelerated anaphylactic response was associated with more rapid histamine‐induced hypovolemia. Mechanistic in vitro and in vivo analyses revealed that endothelial IL‐4Rα PI3K signaling negatively regulates the histamine‐induced endothelial leak response. These results define an unanticipated role for IL‐4Rα–mediated PI3K signaling in negative regulation of IgE‐mediated anaphylactic reactions.

## Introduction

Food allergy is currently on the rise in the Western world: the prevalence of pediatric peanut allergy has doubled from 1997 to 2002 1, 2, 3, 4, and the Centers for Disease Control and Prevention has recently documented an 18% increase in the prevalence of reported food allergy in children from 1997 to 2007 5. Severe food allergy‐related reactions are most often caused by peanuts (50–62%) and tree nuts (15–30%) 6, placing 2.7–5.4 million people at risk for food‐induced anaphylaxis.

A food‐induced anaphylactic reaction encompasses a variety of symptoms that may affect one or more target organs including gastrointestinal, cutaneous, respiratory, and cardiovascular systems 7, 8. Clinical and experimental analyses have identified a central role for IgE/FcϵR/mast cells and mast cell‐derived mediators, including histamine, platelet‐activating factor (PAF), serotonin, proteases (tryptase and chymase), lipid‐derived mediators (prostaglandins [PGD_2_] and leukotrienes [LTC_4,_ LTD_4_, and LTE_4_]), in promoting the clinical manifestations associated with food‐triggered anaphylaxis 9, 10, 11, 12, 13, 14, 15. The interleukin (IL)‐4 /IL‐13 signaling pathway is integral to the food allergic reaction via regulating CD4^+^ Th2 responses, IgE synthesis and mast cell and vascular endothelial cell function 16, 17. Indeed, targeted ablation of IL‐4/IL‐13 signaling alleviates IgE‐mediated, food‐induced allergic reactions 16.

The biological activity of IL‐4 and IL‐13 is regulated via receptor (R) binding: IL‐4 can bind the type I (IL‐4Rα chain and γ_c_ chain) and type II (IL‐4Rα chain and IL‐13Rα1 chain) IL‐4R, and IL‐13 can bind the type II IL‐4R and type II IL‐13R (IL‐13Rα1 and IL‐13Rα2 chains). Ligand (IL‐4 and/or IL‐13) interaction with the type I IL‐4R and type II IL‐4R induces downstream signaling including the signal transducer and activator of transcription (STAT) 6 and phosphatidylinositol 3‐kinase (PI3K) pathways. Phosphorylation of Y575, Y603 and Y633 of human IL‐4Rα mobilizes the transcription factor STAT‐6, which induces IL‐4‐ and IL‐13‐responsive genes 18, 19, 20. Phosphorylation of Y497 of IL‐4Rα, which is part of the IL‐4R motif necessary for recruiting insulin receptor substrate (IRS) 1 and IRS‐2, activates the PI3K and mitogen‐activated protein kinase (MAPK) pathways and mediates IL‐4 proliferative activity 20. Y713 of IL‐4Rα is part of an immunoreceptor tyrosine‐based inhibition motif (ITIM) that binds Src homology 2 (SH2) domain‐containing phosphatases, including SH2 domain‐containing tyrosine phosphatase (SHP) 1 and SHP‐2, and inositol phosphatases and thereby negatively regulates IL‐4/IL‐13 responses 21, 22, 23.

Clinical studies have identified a number of atopic susceptibility genes linking polymorphisms in the IL‐4R/IL‐13 axis with atopic diseases including food allergy and asthma 24, 25. This has been supported by corroborative evidence provided by studies employing mice deficient in components of the IL‐4/IL‐13 signaling pathway and knock‐in murine models demonstrating that disruption of individual signaling domains within the IL‐4Rα in mice can amplify IgE responses and elicits enhanced allergic responses 26, 27, 28. One such mutation is within the part of the IL‐4R motif (Y497 of IL‐4Rα in humans and Y500 of IL‐4Rα in mouse) that regulates PI3K signaling. IL‐4Rα^Y500F^ mice possess a germline mutation in the *Il4ra* gene resulting in a loss of IL‐4Rα‐induced PI3K signaling and leading to impaired IL‐4‐induced CD4^+^ T‐cell proliferation, increased allergen‐induced IgE production and an allergic asthma phenotype 29. In this study, we examined the effects of the IL‐4Rα^Y500F^ mutation on susceptibility of mice to food‐induced anaphylaxis. Unexpectedly, we show that loss of IL‐4Rα‐induced PI3K signaling did not alter susceptibility to IgE‐mediated food‐induced reactions but rather increased histamine‐induced endothelial leak response and accelerated disease progression.

## Materials and Methods

### Animals

Wild‐type (WT) BALB/c and IL‐4Rα^Y500F^ (BALB/c) were originally provided by The Jackson Laboratory, Bar Harbor, ME, USA 29. The mice were crossed to generate heterozygotes (F_1_ IL‐4Rα^Y500F/WT^) and subsequently backcrossed to generate age‐, sex‐, and litter‐matched IL‐4Rα WT and IL‐4Rα^Y500F/Y500F^ mice as described 29. The mice were maintained in a barrier facility, and animals were handled under Institutional Animal Care & Use Committee‐approved protocols from Cincinnati Children's Hospital Medical Center.

### Oral antigen‐induced anaphylaxis

Six‐ to 8‐week‐old mice were sensitized subcutaneously with 50 µg of ovalbumin (OVA) (Sigma–Aldrich, St. Louis, MO, USA) in the presence of 2 mg of aluminum potassium sulfate adjuvant (alum: AIK(SO_4_)_2_‐12H_2_O) (Sigma–Aldrich) in sterile saline. Two weeks later, mice were deprived of food for 5 h and received repeated intragastric (i.g) challenge of OVA (50 mg/250 µL saline) via i.g. feeding needles (Fisher Scientific Co., Pittsburgh, PA, USA). Rectal temperature was monitored at 0, 10, 15, 30, 45, and 60 min following the sixth or seventh OVA challenge with a rectal probe (Physitemp Model BAT‐12) as previously described 30. In some experiments, mice were administered an i.v. (final volume 200 μL) injection with the histamine receptor antagonists Triprolidine (200 μg) and Cimetidine (200 μg) 30 min prior to OVA challenge.

### IL‐4‐ and histamine‐induced hypothermia

Histamine biphosphate monohydrate (Sigma–Aldrich) (25 µg/1 mL saline per mouse) and/or IL‐4C (recombinant, IL‐4‐neutralizing, anti–IL‐4 monoclonal antibody [mAb] complex produced by mixing recombinant mouse IL‐4 with an anti‐IL‐4 mAb [BVD4‐1D11] at a 2:1 molar [1:5 weight] ratio, which saturates the mAb with IL‐4. We have previously demonstrated that these complexes have an in vivo half‐life of approximately 1 day and slowly dissociate, releasing biologically active IL‐4 31. IL‐4C or histamine was i.v. injected, and body temperature was monitored by rectal thermometry every 10 min for 60 min, as we have previously described 30.

### Hematocrit

Blood was drawn from incised mouse tail veins into heparinized capillary tubes and centrifuged for 5 min at 10,000 rpm. Hematocrit (percentage of packed red blood cell [RBC] volume) was calculated as the length of packed RBCs divided by the total length of serum and red cells in the capillary tube and multiplied by 100%, as previously described 15.

### Mast cell quantification

Jejunum (7–10 cm distal to the stomach) were collected and fixed in 10% formalin and processed by standard histologic techniques. Longitudinal sections (5 µm) were stained for mast cells with chloroacetate esterase (CAE) activity, as described previously 30. At least four random sections per mouse per area examined were analyzed. Quantification of stained cells was performed by counting the number of CAE‐positive cells in 5 fields for tongue, 10 fields for ear, and 20 fields for intestine (magnification 400×).

### Enzyme‐Linked Immunosorbent Assay measurements

Mast cell protease 1 (MCPT‐1) serum levels were measured by the mouse MCPT‐1 ELISA Ready‐SET‐Go!, according to the manufacturer's instructions (ebioscience, San Diego, CA, USA). Serum total IgE levels were determined using the ELISA MAX Deluxe SET Mouse IgE Kit (Biolegend, San Diego, CA, USA). Serum OVA‐specific IgE levels were determined by means of ELISA. Plates were coated with anti‐IgE antibody (EM‐95; 10 µg/mL; BD PharMingen, San Jose, CA, USA) and blocked with 200 µL of 10% fetal bovine serum (FBS) before adding serial dilutions of plasma samples (100 µL per well). After overnight incubation, plates were washed and incubated with biotinylated OVA (2.5 mg/mL, 100 µL per well). After 1 h of incubation, streptavidin–horseradish peroxidase (1 mg/mL; Biosource, Camarillo, CA, USA) was added and the assay developed with 100 µL of substrate (TMB substrate reagent set; BD OptEIA, San Diego, CA, USA). Colorimetric reaction was stopped with 1 mol/L H_2_SO_4_ and was quantified by measuring optical density with an ELISA plate reader at 450 nm.

### In vitro permeability

The human vascular endothelial cell line EA.hy926 (ATCC, Manassas, VA, USA) was maintained in DMEM supplemented with 10% FCS, 0.1 mM nonessential amino acids, 1 mM sodium pyruvate, 10 mM HEPES and 1X penicillin/streptomycin (Invitrogen, Grand Island, NY, USA) in a humidified incubator (5% CO_2_, 37°C). On snap wells (12‐mm diameter, 0.4‐µm pore; Corning Glass, Corning, NY, USA), 5 × 10^5^ cells were seeded and cultured for 18–21 days under maintenance media conditions as described above. Transendothelial resistance (TER) was monitored with an EVOM/Endohm (WPI Inc, Sarasota, FL, USA), and endothelial monolayers with TER >100 ohms · cm^2^ were used for all experiments. Monolayers were mounted between the hemi‐chambers of an Ussing apparatus (U2500 dual Ussing chamber, Warner Instruments, Hamden, CT, USA), and 0.112 cm^2^ of tissue was exposed to 10 mL of Krebs buffer at 37°C. The transendothelial potential difference was detected with two paired electrodes that contain 4% agar in 3 M KCl. The electrodes were connected to a voltage clamp amplifier (EC‐800, epithelial voltage clamp, Warner Instruments, Hamden, CT, USA). The electrode potential difference and fluid resistance were compensated before mounting tissue segments into the chamber. To establish equilibrium, potential difference was continuously monitored under open‐circuit conditions for 15 min. Thereafter, the tissues were voltage‐clamped at 0 mV while continuously measuring the short circuit current (*I*_sc_). Voltage pulses (3‐mV square waves sustained for 5 s) were delivered every 50 s to yield a current response for calculation of the resistance across a mucosa from Ohm's law. IL‐4 (10 ng/mL)‐, histamine (100 μM)‐ and vehicle‐stimulated endothelial monolayers were placed in Ussing chambers in the presence and absence of DHEA (100 nM) and allowed to equilibrate for 15 min; basal *I*_sc_ and TER were measured as described previously 21.

### Western Blot analysis

EA.hy926 cell lysates (30 µg) were loaded in 4–12% BisTris gels and transferred to a nitrocellulose membrane (Invitrogen). P85 PI3K was detected by using rabbit polyclonal anti‐p85 PI3K followed by anti‐rabbit peroxidase‐conjugated antibody (Cell Signaling, Danvers, MA) and ECL‐plus detection reagents (GE Healthcare, Pittsburgh, PA). Rabbit anti‐actin (Santa Cruz Biotechnology, Santa Cruz, CA) was used as a loading control.

### Statistical analysis

Data are expressed as mean ± standard deviation (SD), unless otherwise stated. In experiments comparing multiple experimental groups, statistical differences between groups were analyzed using the one‐way ANOVA parametric and a Tukey's multiple comparison post‐test. In experiments comparing two experimental groups, statistical differences between groups were determined using a Student's *t*‐test. *P* <  0.05 was considered significant. Spearman's rank coefficients were used to quantify the relations between hemoconcentration and hypothermia. All analyses were performed with Prism 5.0 software (GraphPad Software Inc., San Diego, CA).

## Results

### Susceptibility of IL‐4Rα^Y500F^ mice to food‐induced anaphylaxis

**Figure 1:**
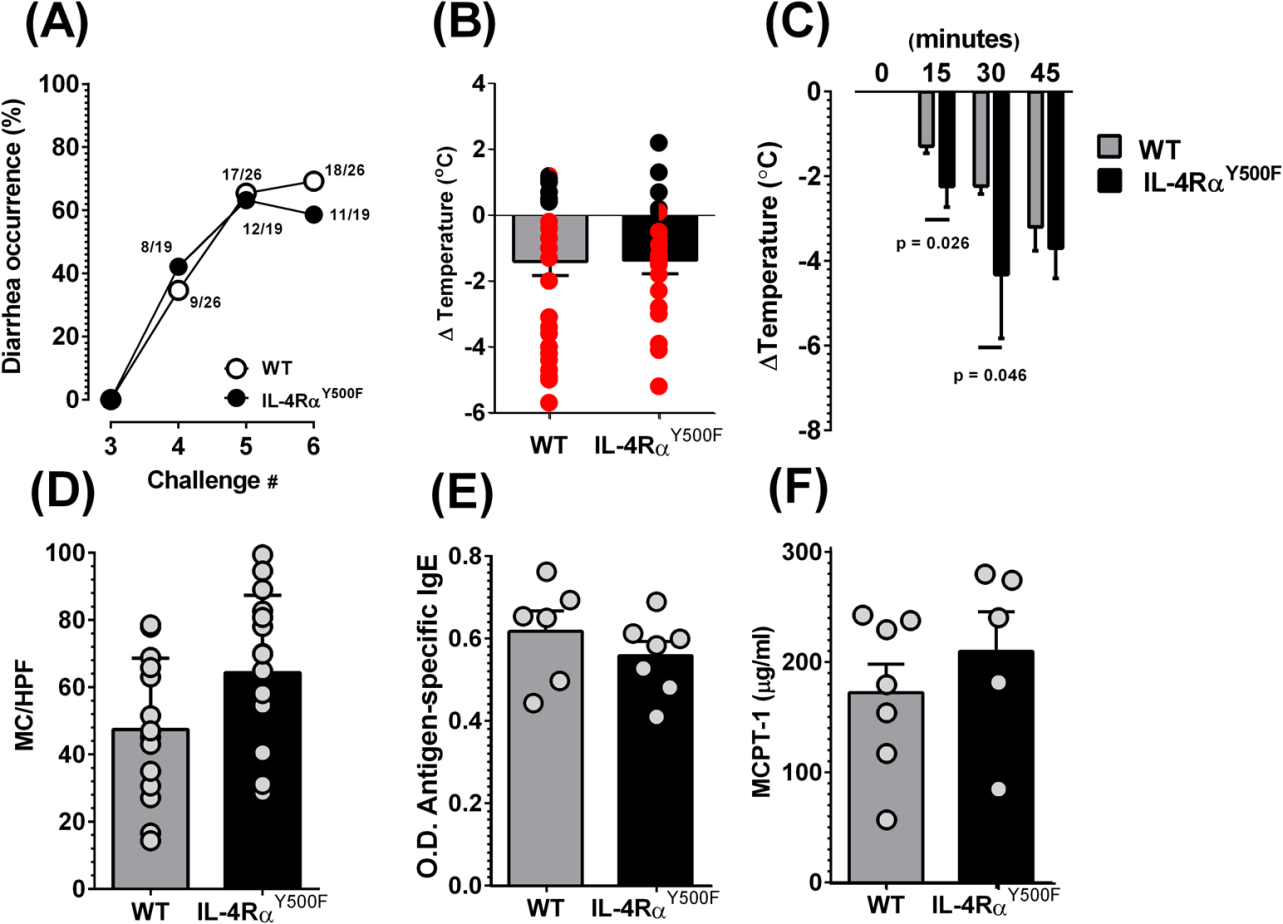
Loss of IL‐4Rα‐PI3K signaling accelerates progression of an anaphylactic reaction. A: Diarrhea occurrence in OVA‐sensitized, intragastric (i.g.) OVA‐challenged WT and IL‐4Rα^Y500F^ mice. B: Temperature change from 0 to 60 min and C: 0, 15, 30, and 45 min following the seventh intragastric (i.g.) OVA challenge in OVA‐sensitized, OVA‐challenged WT and IL‐4Rα^Y500F^ mice. D: Mast cell (MC) numbers per high power field (HPF) in the small intestine, OVA‐specific IgE (E) and mast cell protease 1 (MCPT‐1), (F) levels in the serum of OVA‐sensitized, OVA‐challenged WT and IL‐4Rα^Y500F^ mice following the seventh challenge. A: Data are presented as a percentage of diarrhea occurrence after a number of OVA challenges. The fraction indicates the number of mice with diarrhea out of the total number of mice in that group. (B, D–F: Individual circles represent 1 mouse). B: Red circles represent identification of positive intestinal symptoms of anaphylaxis (diarrhea), and black circles represent no evidence of intestinal symptoms of anaphylaxis. B–F: Data represent mean ± SD; *n* = 4–18 mice per group; *P* values as indicated. O.D., optical density.

Previous studies in the IL‐4Rα^Y500F^ mice have revealed that the Y500F mutation in the IL‐4Rα receptor and loss of IL‐4Rα‐mediated PI3K activation increased allergic inflammation and the asthmatic phenotype 29. To determine the effect of this mutation on susceptibility to food allergy, we assessed intestinal and systemic symptoms of anaphylaxis (diarrhea and hypothermia) in BALB/c WT and IL‐4Rα^Y500F^ mice that were sensitized to OVA and then challenged with OVA via oral gavage 14 days later and then every other day for a total of seven challenges. We observed no significant difference in the occurrence of anaphylaxis between WT and IL‐4Rα^Y500F^ mice (Fig. 1). After the fourth challenge, 34.6% of WT and 42.1% of IL‐4Rα^Y500F^ mice demonstrated symptoms of anaphylaxis, which increased to 77.1% of WT and 84.3% of IL‐4Rα^Y500F^ mice following the seventh challenge (Fig. 1A and B). Assessment of systemic symptom and disease severity (hypothermia) revealed no significant difference in the maximal shock response between WT and IL‐4Rα^Y500F^ mice after the seventh challenge (Fig. 1B). During these analyses, we observed that the IL‐4Rα^Y500F^ mice appeared to develop signs of anaphylaxis earlier than WT mice. Moreover, the IL‐4Rα^Y500F^ mice demonstrated evidence of anaphylaxis (scratching and rubbing around the nose and head, decreased activity with an increasing respiratory rate and pilar erecti) earlier than WT mice following the seventh OVA challenge (results not shown). To quantitate these observations, we examined shock response (body temperature) of the WT and IL‐4Rα^Y500F^ mice at 0, 15, 30, and 45 min after the seventh OVA oral gavage challenge. Indeed, the IL‐4Rα^Y500F^ mice demonstrated a more rapid decrease in body temperature than the WT mice (Fig. 1C). Importantly, by 45 min, there was no significant difference in body temperature between groups (Fig. 1C). These datasets indicate that IL‐4Rα^Y500F^ mice do not have increased susceptibility to food‐induced anaphylaxis or develop a more severe disease phenotype but rather experience an accelerated disease progression.

In previous studies, we have demonstrated that antigen‐specific IgE and intestinal mast cells are the critically important factors in the regulation of food‐induced experimental anaphylaxis 30. Assessing intestinal mast cell levels revealed no differences in number between WT and IL‐4Rα^Y500F^ mice (Fig. 1D). Furthermore, we observed no significant difference in the level of mast cell activation (secreted MCPT 1) or of antigen‐specific and total IgE in WT and IL‐4Rα^Y500F^ mice after the seventh oral gavage challenge (Fig. 1E and F and Fig. S1). We concluded from this that the observed accelerated disease progression cannot be explained by altered IgE and mast cell levels.

**Figure 2:**
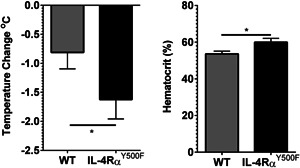
Loss of IL‐4Rα‐PI3K signaling accelerates progression of hypovolemic shock. A: Temperature change from 0 to 10 min and B: percentage hemacrit levels at 10 min following the seventh intragastric (i.g.) OVA challenge in OVA‐sensitized, OVA‐challenged WT and IL‐4Rα^Y500F^ mice. Data represent mean ± SD; *n* = 5–8 mice per group; *P < 0.05.

In mice, the shock organ is the capillary bed; IgE‐mediated, mast cell‐dependent anaphylaxis causes capillary bed dilatation and extravasation, leading to severe hypovolemia 32, 33. A consequence of the hypovolemia‐induced shock in mice is hypothermia 16, 34, 35. Consistent with this concept, we show a direct relationship between hypovolemia (fluid extravasation as measured by hemoconcentration) and severity of oral antigen‐induced anaphylaxis (hypothermia) in our mice (*P* < 0.0001), indicating a direct relationship between fluid extravasation and hypothermia associated with a food‐induced anaphylactic reaction (Fig. S2). To determine whether the increased progression of food allergy in the IL‐4Rα^Y500F^ mice is associated with hypovolemic shock, we examined hypothermia and hemoconcentration in WT and IL‐4Rα^Y500F^ mice 10 min following the seventh oral antigen challenge. We observed a significantly stronger hypothermic response in the IL‐4Rα^Y500F^ mice than WT mice, and the increased temperature loss was associated with increased hemoconcentration, indicating that the IL‐4Rα^Y500F^ mice experience a more accelerated hypovolemic shock response (Fig. 2A and B).

**Figure 3:**
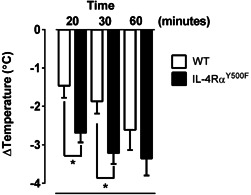
Loss of IL‐4Rα‐PI3K signaling accelerates progression of histamine‐induced hypothermia. Temperature change from 0 to 30 min after i.v. administration of histamine to WT and IL‐4Rα^Y500F^ mice. Data represent *n* = 4 mice per group from three independent experiments and mean ± SD; *P < 0.01.

Previous studies have demonstrated that the systemic manifestations of IgE/mast cell‐dependent anaphylaxis, particularly the hypothermic component of shock response, is mediated by histamine, as it can be blocked by histamine H1 and H2 receptor antagonism 36. Pretreatment of WT and IL‐4Rα^Y500F^ mice with the histamine H1 and H2 receptor antagonist completely abrogated the oral antigen‐induced hypothermia in both WT and IL‐4Rα^Y500F^ mice (Fig. S3), suggesting that the accelerated disease progression in the IL‐4Rα^Y500F^ mice is a consequence of altered histamine‐induced hypothermic response. To determine whether histamine was sufficient to promote accelerated progression of the systemic manifestations of anaphylaxis, naive WT and IL‐4Rα^Y500F^ mice received an i.v. injection of histamine, and hypothermia was assessed. Consistent with our OVA‐induced anaphylaxis experiments, IL‐4Rα^Y500F^ mice experienced an accelerated progression of hypothermia in response to histamine compared to WT mice (Fig. 3). Importantly, we show that administration of equivalent amounts of histamine (25 μg) to WT and IL‐4Rα^Y500F^ mice induced a more accelerated response in IL‐4Rα^Y500F^ mice, suggesting 1) that histamine is sufficient to promote accelerated progression of the shock response in IL‐4Rα^Y500F^ mice and 2) that the observed accelerated response is related in part to altered histamine responsiveness and not histamine levels.

### Increased rate of shock in IL‐4Rα^Y500F^ mice in response to histamine

**Figure 4:**
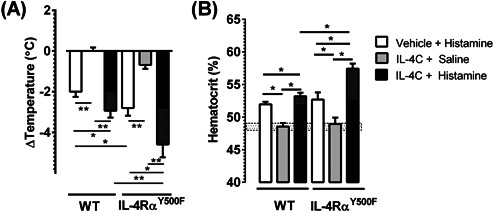
IL‐4 attenuation of histamine‐induced hypothermia is alleviated in IL‐4Rα^Y500F^ mice. A: Temperature change from 0 to 30 min and B: hematocrit at 60 min after i.v. administration of IL‐4C and/or histamine to WT and IL‐4Rα^Y500F^ mice. B: Hatched box indicates average hematocrit level of WT BALB/c mice. Grey checkered box within columns indicates difference between Vehicle + Histamine and IL‐4C + Histamine within the respective strains. Data represent mean ± SD. *n* = 6–18 mice per group. *P < 0.05; **P < 0.01.

It is postulated that histamine‐induced hypothermia is a consequence of vascular endothelial leak and fluid shift into the periphery. Furthermore, previous studies have demonstrated that IL‐4 can modulate histamine‐induced hypothermia 15. The demonstration of 1) a direct link between fluid extravasation and hypothermic response in OVA‐challenged mice; 2) that fluid extravasation and hypothermic response in OVA‐challenged WT and IL‐4Rα^Y500F^ mice were dependent on H1 and H2 receptor and 3) an accelerated hypothermic response in the IL‐4Rα^Y500F^ mice compared to WT mice led us to speculate that the IL‐4Rα/PI3K signaling pathway negatively regulated histamine‐induced vascular endothelial leak. To begin to assess this possibility, we examined the effect of IL‐4Rα^Y500F^ mutation on IL‐4/histamine–induced vascular leak and increased hemoconcentration. WT and IL‐4Rα^Y500F^ mice were primed with IL‐4C and treated 24 h later with the vasoactive mediator histamine. Histamine treatment of WT mice induced a hypothermic response and increased hematocrit, with the former being amplified by pretreatment with IL‐4C (Fig. 4A and B; Average difference between WT Vehicle + Histamine and WT IL‐4C + Histamine: −1.05 ± 0.60 ΔTemperature (°C); mean ± SEM; indicated by gray pattern in column). Similarly, histamine treatment of IL‐4Rα^Y500F^ mice induced hypothermia and increased hematocrit. The temperature change induced by histamine in the IL‐4Rα^Y500F^ mice was significantly greater than that of WT mice (Fig. 4A). Importantly, combined IL‐4C and histamine treatment of IL‐4Rα^Y500F^ mice caused a significantly greater hypothermic response and increase in hematocrit than that observed in histamine only‐treated IL‐4Rα^Y500F^ mice or combined IL‐4C‐ and histamine‐treated WT mice (Fig. 4A and B; *P* < 0.05; Average difference between IL‐4Rα^Y500F^ Vehicle + Histamine and IL‐4Rα^Y500F^ IL4C + Histamine: −2.7 ± 0.66; mean ± SEM; indicated by gray pattern in column). The greater hypothermic and hematocrit response in the absence of PI3K signaling (IL‐4Rα^Y500F^) suggests that IL‐4Rα/PI3K signaling negatively regulates histamine‐induced vascular endothelial responses.

### PI3K signaling negatively regulates histamine‐induced hypothermic response

**Figure 5:**
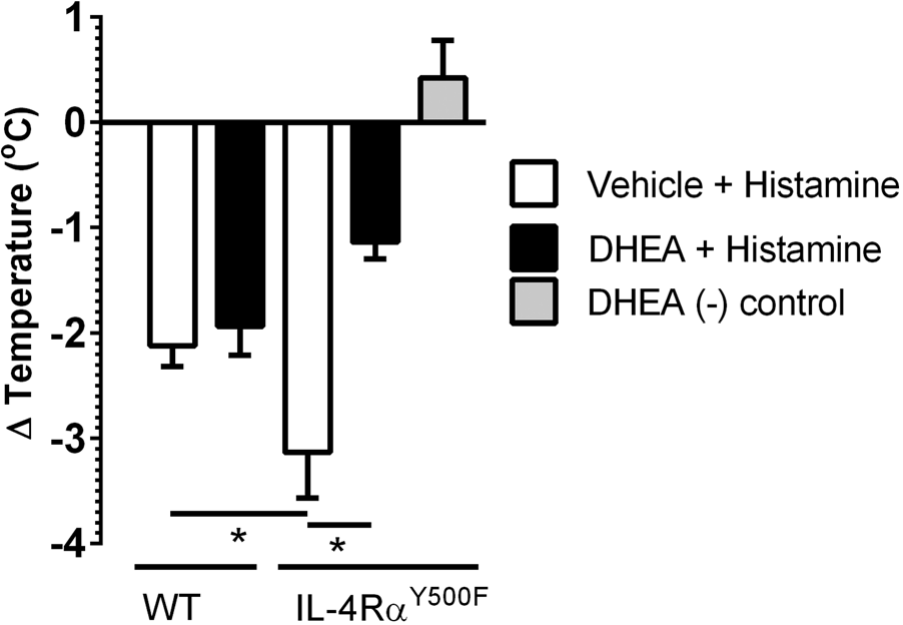
DHEA attenuates histamine‐induced hypothermia in IL‐4Rα^Y500F^ mice. Temperature change from 0 to 30 min after i.v. administration of histamine to WT and IL‐4Rα^Y500F^ mice after pretreatment with vehicle or DHEA (500 μg). Data represent mean ± SD; *n* = 3–8 mice per group from *n* = 2 experiments. *P < 0.01.

The observation of accelerated progression of OVA‐induced and IL‐4/histamine–induced vascular leak indicate that the absence of PI3K signaling (IL‐4Rα^Y500F^) accelerates and/or enhances histamine‐induced vascular endothelial responses. On the basis of these datasets, one would speculate that stimulating vascular endothelial PI3K signaling would attenuate histamine‐induced vascular leak. Dehydroepiandrosterone (DHEA), an adrenal steroid that acts as a precursor in the biosynthesis of testosterone and estrogen, has also been implicated in regulating vascular endothelial cell function 37, 38. Notably, DHEA‐mediated effects are predominantly induced via G‐protein coupled receptor (GPCR)‐stimulated, PI3K/AKT‐dependent activation of FOXO1 37. We therefore speculated that exposure of mice to DHEA would induce vascular endothelial cell PI3K activation and subsequently attenuate histamine‐induced vascular endothelial leak. Firstly, we confirmed that histamine and DHEA stimulation of endothelial cells induce PI3K activation. To do this, we examined phosphorylation of the p85 subunit of PI3K in the human vascular endothelial cell line EA.hy926, which is derived from A549 and HUVEC cells and used as a model of systemic endothelial cells 39, following histamine and DHEA stimulation. We demonstrate increased phosphorylation of the p85 subunit of PI3K between 5 and 15 min following histamine (100 μM) and DHEA (100 nM) stimulation (Fig. S4). Next, WT and IL‐4Rα^Y500F^ mice were pretreated with vehicle or DHEA and IL‐4C and received i.v. histamine treatment 24 h later, after which hypothermia was evaluated. Histamine treatment induced a hypothermic response in WT and IL‐4Rα^Y500F^ mice, with the response being significantly greater in the IL‐4Rα^Y500F^ mice (Fig. 5; *P* < 0.05). Notably, pretreatment with DHEA did not significantly alter the level of hypothermia in histamine‐treated WT mice but did significantly attenuate the level of hypothermia in histamine‐treated IL‐4Rα^Y500F^ mice (Fig. 5; *P* < 0.05). These data suggest that constitutive PI3K activation can attenuate the histamine‐induced increase in hypothermia. Furthermore, these data support the concept that IL‐4–induced PI3K activation attenuates histamine‐induced hypothermia.

**Figure 6:**
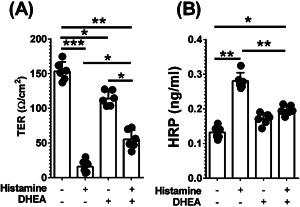
DHEA attenuates histamine‐induced paracellular leak in human vascular endothelial cell line EA.hy926. A: Transendothelial resistance (TER) and B: HRP flux in DHEA‐treated human vascular endothelial cell line (EA.hy926) after histamine stimulation. Confluent (>100 Ω/cm^2^) vascular endothelial cells treated with vehicle or DHEA (100 nM) were stimulated with histamine (100 μM) for 30 min, and TER and HRP flux were determined. Data are representative of 5 wells per treatment group from two independent experiments. Individual circles represent an individual well. Column represents mean ± SD from *n* = 2 experiments. *P < 0.05, **P < 0.01, ***P < 0.005.

As these experiments were performed in IL‐4Rα^Y500F^ global mice, all cells of the hematopoietic and non‐hematopoietic compartment were deficient in IL‐4Rα–mediated PI3K activation. Thus, we cannot determine whether IL‐4Rα–mediated PI3K signaling in endothelial cells directly or indirectly attenuates anaphylactic symptoms. To further elucidate the mechanism, we performed a similar experiment using the human vascular endothelial cell line EA.hy926 39. Histamine stimulation decreases TER of EA.hy926 cells (Fig. 6A). Notably, the decrease in endothelial TER was associated with increased flux of horseradish peroxidase (HRP) (40 kDa), indicating increased paracellular permeability and vascular endothelial leak (Fig. 6B). Stimulation of EA.hy926 cells with DHEA also induced a small decrease in TER and increase in paracellular permeability compared with unstimulated cells (Fig. 6A and B). Importantly, the histamine‐induced increase in endothelial cell permeability was attenuated by pretreatment with DHEA, supporting the concept that endothelial cell PI3K signaling reduces histamine‐induced endothelial permeability.

## Discussion

Previous clinical and murine studies have revealed a link between gain‐of‐function mutations in the IL‐4Rα chain and increased susceptibility to allergic inflammatory responses 26, 27. The majority of the mutations described are thought to drive atopy susceptibility via modulation of the effects of IL‐4/IL‐4Rα on hematopoietic cell function. In this study, we demonstrate that loss of IL‐4Rα/PI3K signaling, via a mutation in the IL‐4R motif necessary for the recruitment of IRS‐1 and IRS‐2, does not increase severity or susceptibility in allergic disease but rather accelerates IgE/mast cell‐mediated, food‐induced anaphylaxis progression in mice. We show that the increased rate of symptom development was not associated with dysregulation of IgE and mast cell function but rather was due to increased sensitivity of the vascular endothelium to mast cell‐derived histamine.

Clinical and murine‐based evidence indicate that the symptoms of food allergy are driven by allergen/IgE/FcϵRI‐mediated mast cell degranulation and release of mast cell mediators that act on target cells to promote the pathophysiologic features of disease, including urticaria, diarrhea, bronchoconstriction, respiratory and cardiovascular collapse, the latter of which reflects a decrease in intravascular volume resulting in decreased vital organ perfusion and shock 10, 11, 14, 15, 40, 41, 42, 43. Consistent with previous reports, we show that the fluid extravasation and decreased intravascular volume (increased hemoconcentration) is dependent on histamine, as pharmacologic antagonism of H1 and H2 receptors inhibited the hypothermic component of shock response 36.

The molecular basis of histamine‐mediated increase in vascular endothelial leak is not yet fully delineated. Histamine ligation to the H1 receptor leads to Gq‐protein–coupled and phospholipase C (PLC) activation, inositol phospholipid hydrolysis and increased intracellular Ca^2+^ 44, 45, which can lead to 1) reduced F‐actin focal attachment formation 46; 2) destabilization of the adherens junction VE‐cadherin and catenin interactions, leading to decreased intercellular tethering resulting in reduced endothelial cell adhesiveness and increased paracellular permeability 47. The IL‐4/IL‐4Rα pathway has previously been shown to magnify the histamine‐mediated effector phase of anaphylaxis 15. The mechanism by which IL‐4 modulates histamine responses is not fully elucidated; however, it is postulated that IL‐4 can magnify histamine responses via enhancement of histamine‐induced PAF synthesis and PGE2 release via IL‐4‐induced upregulation of the H1 receptor expression 48.

Unexpectedly, we show that loss of IL‐4Rα/PI3K signaling leads to an accelerated histamine‐induced hypothermic response and anaphylaxis progression. Murine‐based and in vitro studies indicate that the accelerated response could be attributed to increased responsiveness of the vascular endothelium to histamine. The molecular basis of IL‐4Rα/PI3K‐mediated negative regulation of histamine‐induced anaphylactic shock response is unclear; however, we speculate that the mechanism is related to IL‐4Rα/PI3K's negative regulation of Ca^2+^‐dependent responses. Recent investigations have reported that IL‐4 can attenuate carbachol‐ and caffeine‐induced Ca^2+^ mobilization from the sarcoplasmic reticulum (SR) in airway smooth muscle cells 49. Notably, the IL‐4–mediated inhibition of transient Ca^2+^ release was sensitive to PI3K antagonism, implicating IL‐4–induced PI3K activity in intracellular Ca^2+^ release. Since carbachol‐ and caffeine‐induced Ca^2+^ release in the SR is mediated by different Ca^2+^ release channels, the reduction in the transient Ca^2+^ release by IL‐4/PI3K is not by inhibition of Ca^2+^ release channels but rather by reduction in the amount of SR‐restricted Ca^2+^ levels. Importantly, in some cell types, including HUVECs, PI3K activation promotes PLCγ activation and inositol 1,4,5 triphosphate (IP3) metabolism 50, thus linking IL‐4R activation to PLCγ‐generated IP3 and Ca^2+^ release. In support of PI3K signaling's negative regulation of histamine‐induced shock, we show that constitutive activation of the endothelial PI3K pathway by DHEA attenuated histamine‐induced shock in IL‐4Rα^Y500F^ mice and that DHEA reduced histamine‐induced endothelial paracellular leak in vitro. Demonstrating that DHEA can also suppress histamine responses eliminates concerns with respect to the possibilities of IL‐4Rα^Y500F^ mice possessing an intrinsic defect in endothelial PI3K signaling or IL‐4Rα mediating suppression of Ca^2+^ channel expression or function. Interestingly, we show in vitro that DHEA alone decreased endothelial barrier function as compared with unstimulated endothelial cells. Notably, this baseline DHEA‐induced effect was not related to increased paracellular leak, as there were no differences in HRP flux between vehicle‐treated and DHEA only‐treated cells, suggesting that DHEA was stimulating altered ion secretion.

Previous studies in IL‐4Rα^Y500F^ mice have demonstrated a role for the loss of IL‐4Rα/PI3K signaling in the exacerbation of allergic inflammatory responses 29. Moreover, in a pulmonary airway inflammation model, IL‐4Rα^Y500F^ mice developed a more severe asthmatic phenotype as demonstrated by increased airway hyperresponsiveness, pulmonary eosinophilia and mucus hypersecretion 29. We did not observe increased severity of food‐induced anaphylaxis but rather the accelerated rate of symptom onset in IL‐4Rα^Y500F^ mice. Importantly, the features of pulmonary allergic inflammation in this particular murine asthma model are not dependent on mast cell–mediated vascular endothelial permeability and fluid extravasation. However, Blaeser et al. reported that the IL‐4Rα^Y500F^ mice had increased total IgE and allergen‐induced IgE production following OVA/Alum immunization 29. In contrast, we did not observe differences in total and antigen‐specific IgE following OVA/Alum immunization and challenge. One possible explanation for the observed differences between our studies and that of Blaeser et al. with respect to serum IgE is the intestinal microbial diversity 51. Recent mouse studies indicate that absence of microbial colonization or colonization with low‐diversity microbiota leads to increased serum IgE levels and enhancement of CD4^+^ T‐cell and IL‐4 responses 51.

We show that though MCPT‐1 levels were comparable between WT and IL‐4Rα^Y500F^ mice 60 min following OVA challenge, IL‐4Rα^Y500F^ mice experienced an accelerated progression of hypovolemia and hypothermia compared to WT mice, suggesting that the IL‐4/PI3K pathway alters histamine‐mediated responses. We cannot rule out the possibility of a more rapid activation of mast cells and increase in the level of mast cell mediators in the IL‐4Rα^Y500F^ mice, which could accelerate progression of the oral antigen‐induced anaphylactic symptoms. Consistent with this argument, IL‐4 has been shown to amplify mast cell secretory function and release of preformed mediators such as serotonin and arachidonates 52, 53. However, we do show that administration of 25 μg of histamine to IL‐4Rα^Y500F^ mice also lead to an accelerated progression of hypovolemia and hypothermia, suggesting that the altered response in IL‐4Rα^Y500F^ mice can be attributed in part to altered sensitivity of the vascular endothelium to mast cell‐derived histamine.

A number of murine‐based studies have revealed that additional gain‐of‐function mutations in IL‐4Rα can enhance allergic inflammatory responses. IL‐4Rα^Y709F^ mice, which have a tyrosine to phenylalanine mutation at position 709 within the ITIM of IL‐4Rα, have increased susceptibility to allergen‐induced airway inflammation and enhanced sensitivity to food allergens 26, 27. Similarly, mice that possess the glutamine to arginine substitution at position 576 (Q576R) of IL‐4Rα exhibited increased allergen‐induced inflammation and remodeling 28. To the best of our knowledge, this study is the first demonstration that an IL‐4Rα mutation can accelerate disease progression. Though no polymorphisms have been observed in the human equivalent tyrosine residue within the insulin: IL‐4 receptor motif (Y497), a human serine proline polymorphism six amino acids downstream of Y497 and within the IL‐4Rα ITIM motif (S503P) has been reported 54, 55. The impact of this polymorphism on the function of the PI3K motif of the human IL‐4Rα chain is currently unknown; however, the presence of polymorphic amino acid residues at this location (P503 and R576) are known to alter receptor polarity and secondary structure and affect IRS‐1 and IRS‐2 propagation of the IL‐4Rα signaling 54.

In the current manuscript, we show that loss of IL‐4Rα–mediated PI3K signaling accelerates the progression of oral antigen‐induced anaphylactic reactions. In vitro and in vivo studies suggest that IL‐4Rα PI3K signaling negatively regulates histamine‐mediated vascular endothelial leak and loss of this pathway leads to accelerated histamine‐mediated hypovolemic shock and hypothermia. These results define an unanticipated role for IL‐4Rα–mediated PI3K signaling in negative regulation of IgE‐mediated anaphylactic reactions.

## Disclosures

All of the authors have declared that they have no conflict of interest.

## Supporting information

Additional supporting information may be found in the online version of this article at the publisher's web‐site.

**Figure S1**. No effect of loss of IL‐4Rα‐PI3K signaling on total IgE. Total IgE levels in the serum of OVA‐sensitized, intragastric (i.g.) OVA‐challenged WT and IL‐4Rα^Y500F^ mice following the seventh challenge. Each filled circle represents an individual mouse. Data represent mean ± SD.
**Figure S2**. A positive relationship between vascular leak and shock response in murine oral antigen‐induced anaphylaxis. Correlation between hematocrit and systemic symptoms of oral antigen‐induced anaphylaxis. Spearman's rank correlation coefficient between hematocrit and temperature change from 0 to 60 min after the seventh intragastric (i.g.) OVA challenge in OVA‐sensitized WT mice. Individual symbols represent 1 mouse.
**Figure S3**. Systemic anaphylaxis in WT and IL‐4Rα^Y500F^ mice is dependent on histamine. Temperature change from 0 to 30 min in OVA‐sensitized, intragastric (i.g.) OVA‐challenged (A) WT and (B) IL‐4Rα^Y500F^ mice following the sixth and seventh intragastric (i.g.) OVA challenge. OVA‐sensitized WT and IL‐4Rα^Y500F^ mice receive repeated i.g. OVA challenges, and temperature change from 0 to 30 min was determined following the sixth challenge. Prior to the seventh challenge, mice were administered the histamine Type 1 and type 2 receptor antagonists Triprolidine (200 μg) and Cimetidine (200 μg) intravenously (i.v.) (200 μL final volume) 30 min prior to OVA challenge. Each filled circle represents an individual mouse. Data represent the temperature change from 0 to 30 min following the sixth and seventh challenge; *P* values as indicated.
**Figure S4**. Histamine and DHEA‐induced PI3K activation in human vascular endothelial cell line EA.hy926. Representative Western blot analyses probing for PI3K p85 full‐length protein and actin in protein lysates from human vascular endothelial cell line EA.hy926 following 0, 1, 5, 15, 30 and 60 min stimulation with histamine (20 nM) or DHEA (100 nM).
