# Supplementary material for: Loss of IL‐4Rα–mediated PI3K signaling accelerates the progression of IgE/mast cell–mediated reactions
Source: Immun Inflamm Dis. 2015 Sep 17;3(4):420–30. doi: 10.1002/iid3.80 (PMC4693723; doi:10.1002/iid3.80)

Loss of IL-4Rα–mediated PI3K signaling accelerates the onset and progression of IgE/mast cell–mediated reactions

Jane Sledd*, David Wu*, Richard Ahrens*, Jeebong Lee*, Lisa Waggoner*, Ying Ting Tsai*, Yui-Hsi Wang*, Simon P. Hogan*

* Division of Allergy and Immunology and Immunobiology, Department of Pediatrics, University of Cincinnati College of Medicine, Cincinnati Children's Hospital Medical Center, , 3333 Burnet Avenue, Cincinnati, OH, 45229

Supplementary Figure S1. No impact of loss of IL-4Rα-PI3K signaling on total IgE. Total IgE levels in the serum of OVA-sensitized, intragastric (i.g.) OVA-challenged WT and IL-4Rα^{Y500F} mice following the seventh challenge. Each filled circle represents an individual mouse. Data represents mean ± SD.

Supplementary Figure S2. A Positive relationship between vascular leak and shock response in murine oral antigen-induced anaphylaxis. Correlation between hematocrit and systemic symptoms of oral antigen–induced anaphylaxis. Spearman's rank correlation coefficient between hematocrit and temperature change from 0 to 60 minutes after the seventh i.g. OVA challenge in OVA-sensitized WT mice. Individual symbols represent 1 mouse.

Supplementary Figure S3. Systemic anaphylaxis in WT and IL-4Rα^{Y500F} mice is dependent on Histamine. Temperature change from 0 to 30 minutes in OVA-sensitized, intragastric (i.g.) OVA-challenged (A). WT and (B). IL-4Rα^{Y500F} mice following the sixth and seventh intragastric (i.g.) OVA challenge. OVA-sensitized WT and IL-4Rα^{Y500F} mice receive repeated i.g. OVA challenges and Temperature change from 0 to 30 minutes was determined following the sixth challenge. Prior to the seventh challenge mice were administered histamine Type 1 and Type 2 receptor antagonists Triprolidine (200μg) and Cimetidine (200μg) i.v. (200μl final volume) 30 minutes prior to OVA challenge. Each filled circle represents an individual mouse. Data represents the temperature change from 0 to 30 minutes following the sixth and seventh challenge; p values as indicated.

Supplementary Figure S4. Histamine and DHEA-induced PI3K activation in human vascular endothelial cell line EA.hy926. Representative Western blot analyses probing for PI3K p85-full-length-protein and actin in protein lysates from human vascular endothelial cell line EA.hy926 following 0, 1, 5, 15, 30 and 60 minutes stimulation with Histamine (20nM) or DHEA (100nM).

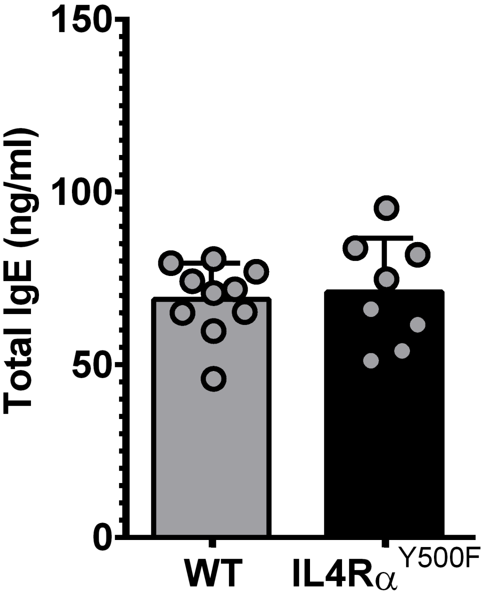

Supplemental Figure S1.

Supplemental Figure S2

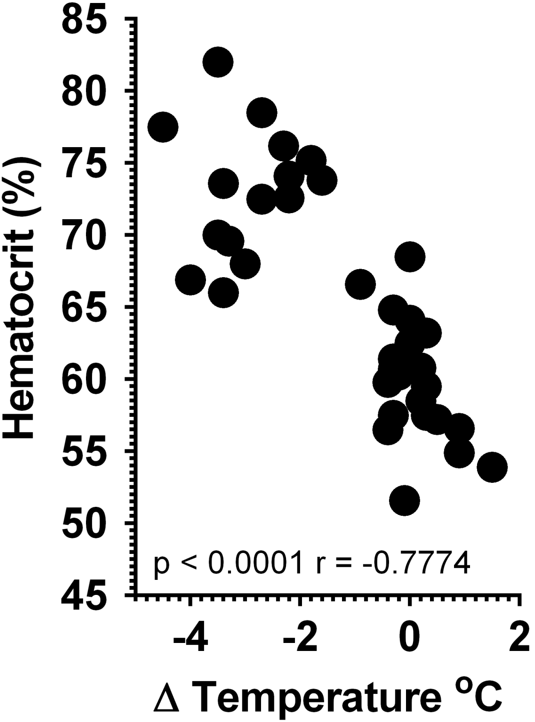

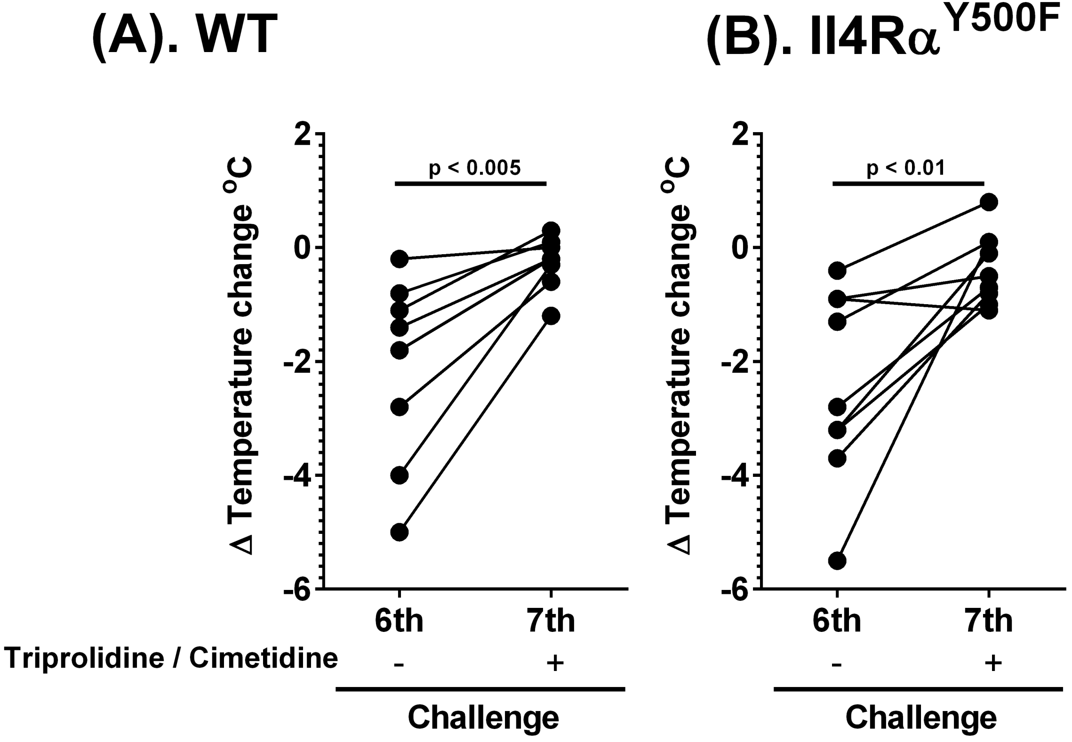

Supplemental Figure S3.

Supplemental Figure S4

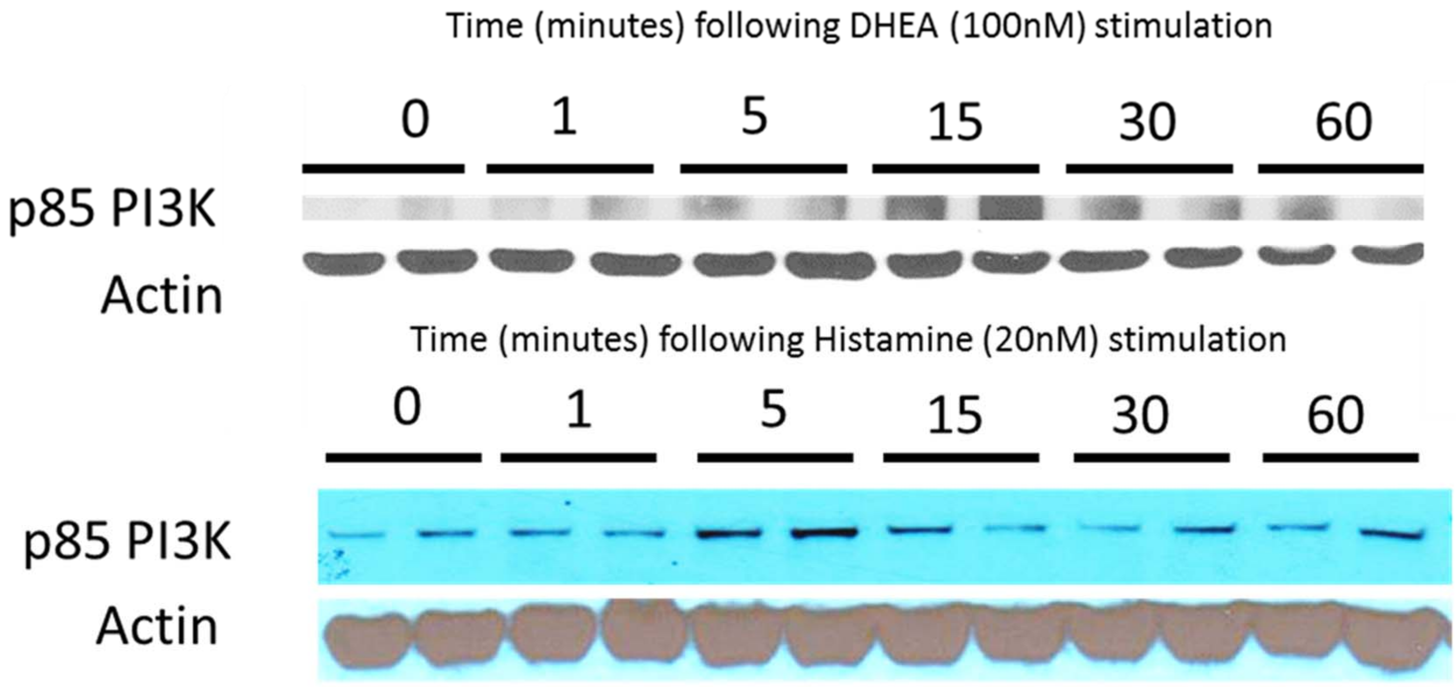

Supplement: Supplementary file 1 — Figure S1. No effect of loss of IL‐4Rα‐PI3K signaling on total IgE. Total IgE levels in the serum of OVA‐sensitized, intragastric (i.g.) OVA‐challenged WT and IL‐4RαY500F mice following the seventh challenge. Each filled circle represents an individual mouse. Data represent mean ± SD. Figure S2. A positive relationship between vascular leak and shock response in murine oral antigen‐induced anaphylaxis. Correlation between hematocrit and systemic symptoms of oral antigen‐induced anaphylaxis. Spearman's rank correlation coefficient between hematocrit and temperature change from 0 to 60 min after the seventh intragastric (i.g.) OVA challenge in OVA‐sensitized WT mice. Individual symbols represent 1 mouse. Figure S3. Systemic anaphylaxis in WT and IL‐4RαY500F mice is dependent on histamine. Temperature change from 0 to 30 min in OVA‐sensitized, intragastric (i.g.) OVA‐challenged (A) WT and (B) IL‐4RαY500F mice following the sixth and seventh intragastric (i.g.) OVA challenge. OVA‐sensitized WT and IL‐4RαY500F mice receive repeated i.g. OVA challenges, and temperature change from 0 to 30 min was determined following the sixth challenge. Prior to the seventh challenge, mice were administered the histamine Type 1 and type 2 receptor antagonists Triprolidine (200 μg) and Cimetidine (200 μg) intravenously (i.v.) (200 μL final volume) 30 min prior to OVA challenge. Each filled circle represents an individual mouse. Data represent the temperature change from 0 to 30 min following the sixth and seventh challenge; P values as indicated. Figure S4. Histamine and DHEA‐induced PI3K activation in human vascular endothelial cell line EA.hy926. Representative Western blot analyses probing for PI3K p85 full‐length protein and actin in protein lysates from human vascular endothelial cell line EA.hy926 following 0, 1, 5, 15, 30 and 60 min stimulation with histamine (20 nM) or DHEA (100 nM). [file IID3-3-420-s001.pdf]
